# Synaptic Spinules in the Olfactory Circuit of *Drosophila melanogaster*

**DOI:** 10.3389/fncel.2018.00086

**Published:** 2018-03-27

**Authors:** Lydia Gruber, Jürgen Rybak, Bill S. Hansson, Rafael Cantera

**Affiliations:** ^1^Department of Evolutionary Neuroethology, Max Planck Institute for Chemical Ecology (MPG) Jena, Germany; ^2^Departamento de Biología del Neurodesarrollo, Instituto de Investigaciones Biológicas Clemente Estable (IIBCE) Montevideo, Uruguay; ^3^Zoology Department, Stockholm University Stockholm, Sweden

**Keywords:** olfactory circuitry, *Drosophila melanogaster*, synaptic spinules, FIB-SEM, synaptic plasticity

## Abstract

Here we report on ultrastructural features of brain synapses in the fly *Drosophila melanogaster* and outline a perspective for the study of their functional significance. Images taken with the aid of focused ion beam-scanning electron microscopy (EM) at 20 nm intervals across olfactory glomerulus DA2 revealed that some synaptic boutons are penetrated by protrusions emanating from other neurons. Similar structures in the brain of mammals are known as synaptic spinules. A survey with transmission EM (TEM) disclosed that these structures are frequent throughout the antennal lobe. Detailed neuronal tracings revealed that spinules are formed by all three major types of neurons innervating glomerulus DA2 but the olfactory sensory neurons (OSNs) receive significantly more spinules than other olfactory neurons. Double-membrane vesicles (DMVs) that appear to represent material that has pinched-off from spinules are also most abundant in presynaptic boutons of OSNs. Inside the host neuron, a close association was observed between spinules, the endoplasmic reticulum (ER) and mitochondria. We propose that by releasing material into the host neuron, through a process triggered by synaptic activity and analogous to axonal pruning, synaptic spinules could function as a mechanism for synapse tagging, synaptic remodeling and neural plasticity. Future directions of experimental work to investigate this theory are proposed.

## Introduction

Research conducted in evolutionarily distant animals has contributed to our current understanding of olfactory synaptic circuits (Hildebrand and Shepherd, 1997; Ache and Young, 2005). The olfactory neuronal circuitry of the fly *Drosophila melanogaster* has been investigated successfully with anatomical, physiological, genetic and behavioral approaches and good models have been proposed to understand how chemosensory information is processed and how olfactory circuits contribute to learning and memory (Davis, 2004; Keene and Waddell, 2005; Fiala, 2007; Wilson, 2013; Guven-Ozkan and Davis, 2014; Hige, 2017).

This bounty of knowledge stood until recently in bright contrast to our insufficient understanding of the synaptic connections formed between the different cellular components of the olfactory neuronal network. Because of the small size of synapses and the need to map them in 3D across relatively large volumes of brain tissue, electron microscopy (EM) is necessary to map all synapses of the olfactory circuit. Progress in volume-based EM, image analysis, and automatic 3D reconstruction facilitates this challenging task and makes it possible to image and analyze all synaptic sites in the volume spanning the region of interest (Helmstaedter, 2013; Schneider-Mizell et al., 2016; Zheng et al., 2017). These recent advances have already resulted in several publications reporting detailed information on olfactory microcircuits in *Drosophila* (Berck et al., 2016; Rybak et al., 2016; Takemura et al., 2017; Tobin et al., 2017).

We used focused ion beam-scanning EM (FIB-SEM; Knott et al., 2008) to acquire complete series of images taken at 20 nm intervals across the entire olfactory DA2 glomerulus in adult *Drosophila* females (Gruber et al., unpublished data). The ultimate goal is to obtain a complete connectome of this glomerulus, which plays an important ecological role since it senses the odorant geosmin, emitted by mold growing in rotten fruits, and mediates a life-saving escape in the fly (Stensmyr et al., 2012). In the course of our studies we observed that olfactory neurons form deep invaginations of their plasma membrane nearby synaptic sites, occupied by protrusions from other neurons, similar to what has been referred to as synaptic spinules in the mammalian brain and that had yet not been reported for *Drosophila*. Synaptic spinules are invaginating protrusions of variable size and morphology that penetrate presynaptic terminals and, less frequently, postsynaptic profiles, axons and even glia in the brain of mammals and other vertebrates (reviewed in Petralia et al., 2015). Synaptic spinules are dynamic structures that grow and proliferate following synaptic activity (Richards et al., 2005; Tao-Cheng et al., 2009) and have been suggested to contribute to membrane plasticity as well as to cell-to-cell communication and material exchange between neurons in an activity-dependent fashion (Petralia et al., 2015).

Our knowledge of these synapse-associated structures is still very limited. Here we present a viewpoint on this subject. We predict that spinules mediate localized synaptic plasticity mainly among olfactory sensory neurons (OSNs). Thus the finding of synaptic spinules in *Drosophila melanogaster* opens an avenue for an experimental investigation of their contribution and relevance for synapse plasticity, benefiting from the exceptional advantages offered by this organism.

## Results and Discussion

**Figure 1:**
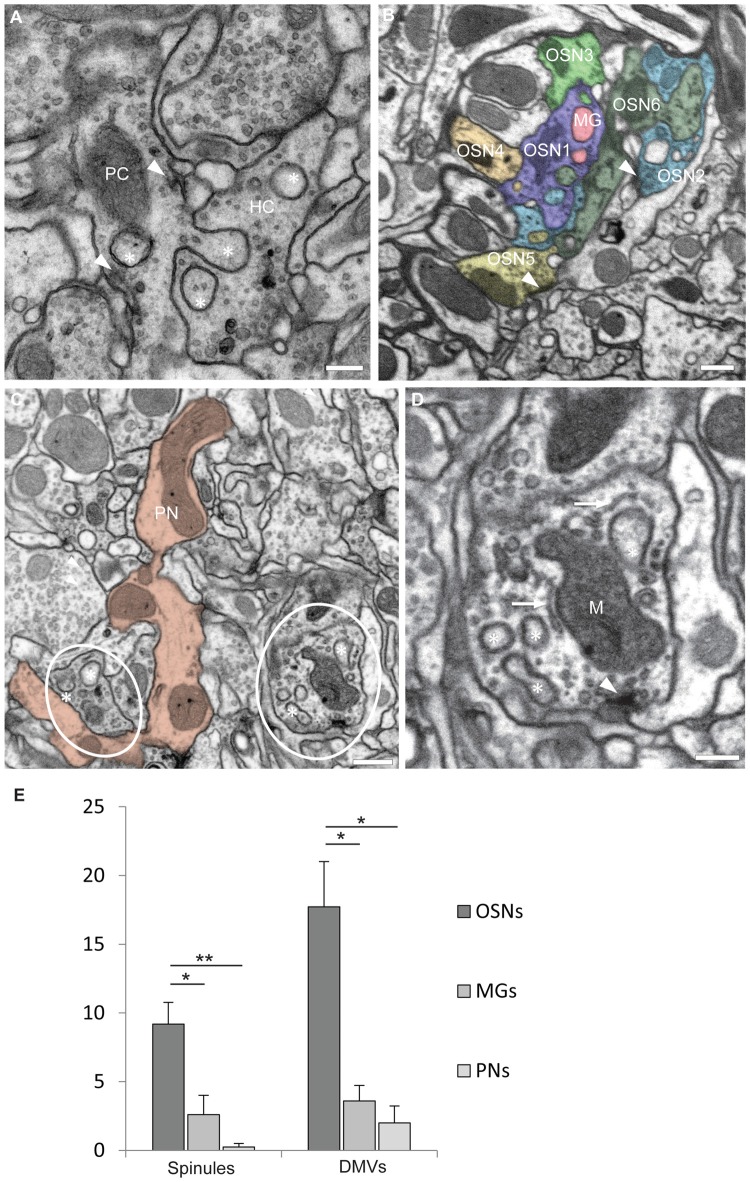
Olfactory neurons in glomerulus DA2 contain invaginating protrusions. **(A)** Transmission electron microscopy (TEM) image of a 50 nm section showing examples of invaginating protrusions, or spinules (asterisks), enclosed by two plasma membranes and close to presynaptic sites (arrowheads). Notice that the evaginating membrane of the protruding cell (PC) is tightly adjoined by the invaginating membrane of the host cell (HC). Scale bar = 200 nm. **(B)** A synaptic bouton can receive invaginating protrusions from more than one neuron. This image from a focused ion beam-scanning EM (FIB-SEM) serial reconstruction of glomerulus DA2 depicts invaginating protrusions in presynaptic boutons of two different olfactory sensory neurons (OSN1 and OSN2) penetrated by protrusions from several neighboring cells. The PCs in this particular example are either a multiglomerular neuron (MG) or other OSNs (OSN3, 4, 5 and 6). Synaptic sites are indicated by an arrowhead and the reconstructed neurons are color-coded to assign the origin of the invaginated protrusions inside HCs. Scale bar = 500 nm. **(C)** Invaginating protrusions are not equally abundant among different types of olfactory neurons. This image (FIB-SEM) shows for example several boutons (red) of a uniglomerular projection neuron (PN), devoid of protrusions. In contrast, nearby OSN boutons (encircled) contain several protrusions (asterisks; see quantification in **E**). Scale bar = 500 nm. **(D)** FIB-SEM image showing invaginating protrusions (asterisks) close to mitochondria (M), putative endoplasmic reticulum (ER) cisternae (arrow) and a presynaptic site (arrowhead). For 3D surface view of spinules see Figure 2B. Scale bar = 200 nm. **(E)** Quantification of spinules and double-membrane vesicles (DMVs) found inside reconstructed OSNs (*n* = 11), Projection neurons (PNs) (*n* = 4) and MGs (*n* = 5). OSNs receive a larger number of spinules and DMVs compared to MGs and PNs. Quantification was done in one brain. Mean values with standard error of the mean are depicted. *p < 0.1; **p < 0.01, one-way ANOVA, Tukey *post hoc* test.

The observations reported here were done in the antennal lobe of female adults of *Drosophila melanogaster* studied with transmission electron microscopy (TEM, five specimens) and FIB-SEM (two specimens) across the entire DA2 glomerulus (see Supplementary Material). To achieve serial sections of this particular region with FIB-SEM it was marked previously by fiducial laser marks (see Supplementary Material). Images revealed that olfactory neurons make an interdigitating system of invaginating protrusions 20–500 nm in diameter close to active sites. Protrusions, emanating from one synaptic partner (the “protruding cell, PC”), penetrate the narrow funnels formed by deep invaginations of the plasma membrane of another synaptic partner (the “host cell, HC”; Figures 1A,B). The protrusions are therefore covered by two membranes: the evaginated membrane of the PC tightly covered by the invaginated membrane of the HC, which receives the protrusion (Figure 1A). FIB-SEM-based dense reconstructions (done with the TrakEM2 plugin for ImageJ Fiji^1^; see Supplementary Material) make it possible to study invaginating protrusions in different types of olfactory neurons, which were distinguished according to their morphology (branching pattern and diameter of single branches), their total volume inside one glomerulus and ultrastructural details (as for example their synaptic inventory of input and output synapses) and other criteria described previously (Rybak et al., 2016; Tobin et al., 2017). These criteria allow a clear identification of uniglomerular projection neurons (PNs) and olfactory receptor neurons (OSNs) whereas the remaining cell types were more difficult to distinguish and are described here with the generic term “multigomerular neurons” (MGs). Individual presynaptic boutons of olfactory neurons might receive protrusions from more than one neuron or cell type, most prominently seen in OSNs (Figure 1B), and mutually invaginating protrusions between two neurons were also observed (not shown) as reported previously for other olfactory glomeruli (Rybak et al., 2016; in Figures 5C,D). Many of invaginating protrusions traced to their fiber of origin were found to originate from other OSNs, whereas the remaining ones emanated either from MGs, which includes local interneurons and multiglomerular PNs (Figure 1B), or PNs (see Figures 5C,D in Rybak et al., 2016). The synaptic boutons of PNs were mostly devoid of protrusions (Figures 1C,E).

By size, shape and location these invaginating protrusions are interpreted here to be the type of structures which in mammalian brain have been designated as synaptic spinules (Petralia et al., 2015). They appear to be identical or very similar to invaginated profiles illustrated in images of *Drosophila* synapses in other brain neurons published by other authors, who did not name them explicitly (see for example Figures 4A,B in Leiss et al., 2009; Figure 3 in Butcher et al., 2012; Figure 1 in Berck et al., 2016; Figures 6D and Supplementary Figure S1A in Zheng et al., 2017). Our survey of several *Drosophila* brains with the aid of TEM confirmed that spinules are frequent throughout the antennal lobe (data not shown).

**Figure 2:**
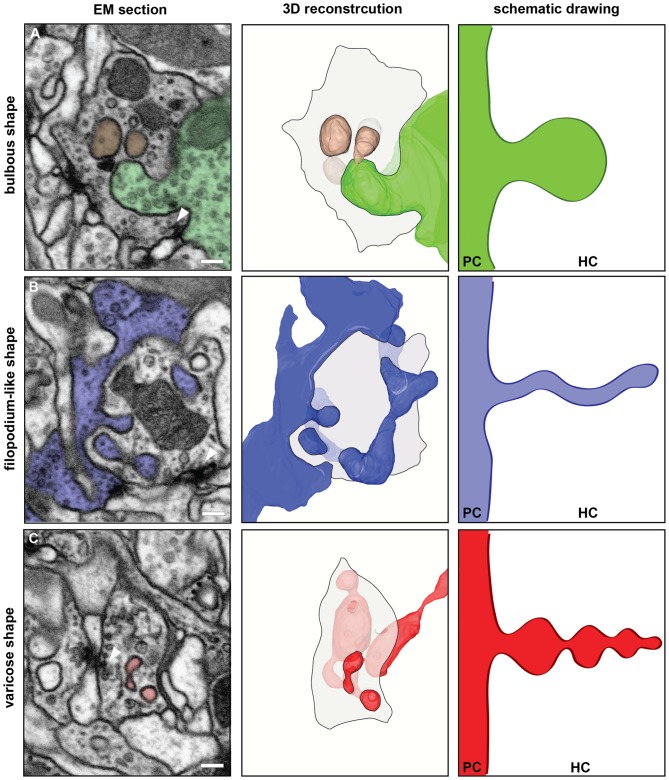
FIB-SEM based 3D reconstructions of synaptic spinules disclosed their morphological diversity. The left column shows single images from the FIB-SEM-series used for the 3D reconstructions illustrated in the middle column. The reconstructed spinules and their cells of origin are colored in green **(A)**, blue **(B)** or red **(C)** DMVs are shown in brown **(A)**. Spinules and DMVs are close to presynaptic sites (arrowheads). Scale bar = 200 nm. The middle column shows surface views of the 3D reconstructions with the same color code. The HC is illustrated by a transparent shaded area, representing one section plane of the HC neurite, outlined with a black line. The right column shows schematic drawings of the different morphological types of spinules, emanating from the PC and growing into the HC, to illustrate the morphological diversity of synaptic spinules found here in glomerulus DA2. **(A)** The HC is an OSN containing a bulbous spinule (green) and two DMVs (brown) most likely pinched-off from the spinule. **(B)** shows an example of a filopodium-like spinule (blue) and **(C)** a varicose spinule (red).

The spinules reported here contained cytoplasm and in many cases also clear and dark vesicles (Figures 1A,D, 2A,B). The size of the spinules and that of their host boutons imply that spinules are in close vicinity with other organelles. Practically all spinules were observed in the proximity of presynaptic sites (Figures 1, 2), mitochondria and what appeared to be cisternae of the endoplasmic reticulum (ER) of the HC (Figure 1A,C,D). In many cases spinules appeared to be in physical contact with mitochondria and ER. Therefore, spinules might be part of a recently well described neuronal ER network that includes contacts with the plasma membrane, mitochondria as well as lysosomes and multivesicular bodies (Wu et al., 2017). Similar connections between ER tubules and synaptic invaginations have been observed previously in presynaptic regions of visual receptor cells (Lovas, 1971). The close association between spinules, active sites and two major sources of Ca^2+^ might have functional consequences.

A quantification of every single spinule penetrating each randomly selected HC of each neuronal type (in one brain) inside glomerulus DA2 indicated that OSNs receive spinules most frequently, MGs less frequently and PNs only rarely (Figure 1E). On the other hand, based on EM images published by others (Leiss et al., 2009; Butcher et al., 2012) we propose that PN presynaptic boutons, located in the calyx of the mushroom body, host abundant spinules protruding from their postsynaptic partners, the Kenyon cells.

The shape of spinules appeared to be variable. They were often relatively short and bulbous (Figure 2A) but sometimes more elongated, filopodium-like (Figure 2B) or varicose (Figure 2C) and even branched (Figure 2C). Inside their HCs synaptic spinules were closely associated with cellular entrapments of similar appearance and size, but not connected to other neurons and thus entirely embedded in the cytoplasm of the HC (Figure 2A). Similar to what is reported above for spinules, the two membranes in these “disconnected” profiles enclosed a cytosolic content with vesicles (Figure 2A). At synapses in the vertebrate brain, profiles of this type are called “double-membrane vesicles” (DMVs) and are considered to pinch-off from spinules (see for example Spacek and Harris, 2004; reviewed in Petralia et al., 2015). A quantification of DMVs in randomly selected host neurons (same as for spinule quantification) among the DA2 in one *Drosophila* brain (see Supplementary Material) revealed that, just like spinules, these structures are most abundant inside OSNs (Figure 1E), thus reinforcing the idea that they are derived from spinules. These vesicles appear to us to be clearly distinct from exosomes and other types of extracellular vesicles used by a variety of cell types and tissues to communicate at a distance through exchange of protein and RNA (Cocucci and Meldolesi, 2015; Budnik et al., 2016) secreted into the extracellular space with consequences for synaptic maintenance, plasticity and homeostasis (Korkut et al., 2009; Budnik et al., 2016; Ashley et al., 2018). A major difference between exosomes and the DMVs reported here is that the latter are delivered directly into the cytoplasm of the HC, enabling modification of the function of individual synapses, without affecting the function of other synapses of the same neuron.

The observation that some of the spinules observed in our reconstructed volume of glomerulus DA2 had a varicose shape might be relevant for a speculative interpretation of their functions. In *Drosophila*, during its metamorphosis from larva to adult, axonal and dendritic fibers become first varicose and subsequently subdivide into fragments in a process known as pruning, which is controlled by the steroid hormone ecdysone and triggered by Ca^2+^ (Yaniv and Schuldiner, 2016). We propose that in adult olfactory circuits synaptic-activity induced release of Ca^2+^ from mitochondria and ER, observed here to be in close proximity and contact to spinules at synaptic sites, could induce not only spinule growth and proliferation as previously proposed (Richards et al., 2005; Tao-Cheng et al., 2009; Ueda and Hayashi, 2013) but also spinule fragmentation inside the host neuron through a process analogous to the pruning of axonal terminals and dendritic branches during metamorphosis, with the difference that in this case the fragments are generated intracellularly and become DMVs in the HC.

It has been suggested that synaptic spinules mediate trans-synaptic exchange of material (reviewed in Petralia et al., 2015). Hence, activity-triggered spinule fragmentation mainly in OSNs could be the basis for localized synaptic plasticity, mediated by transference between synaptic partners of microRNA, proteins or other material (Edelstein and Smythies, 2014; Smalheiser, 2014; Busto et al., 2017) and affecting only one synaptic bouton of dozens present among the branches of a given neuron. This localized transference of material between OSNs and other neurons, at individual synaptic boutons that receive spinules and DMVs, could also mediate propagation of epigenetic changes and other modifications. It has been shown that spinules formation is induced by artificial generation of LTP (Toni et al., 1999; Stewart et al., 2005; Ueda and Hayashi, 2013). Concurrent synaptic activity dependent fragmentation of spinules could therefore be involved in synapse tagging and capture (Frey and Morris, 1997; Redondo and Morris, 2011) and would have functional consequences for future synaptic activity, including olfactory learning and memory processes.

*Drosophila* *melanogaster*, as a model organism, opens an avenue for future experimental investigations of the ideas outlined here. In a short perspective, experiments should be designed to demonstrate in a more conclusive way that the DMVs reported here are derived from the spinules and that this involves fragmentation of the spinules. Appropriate combinations of genetic labeling of pre- and postsynaptic neurons with different fluorophores and super resolution microscopy can be used for this aim. Screens of genetically tagged marker proteins or RNA, synthetized exclusively by one neuronal type and that ends up inside neurons which do not express the marker, would prove the exchange of material. Furthermore, decrease in activity-dependent spinule formation and fragmentation after blockage of mitochondrial Ca^2+^ release would prove our suggestion of this interplay.

Exchange of material via DMVs might serve synaptic tagging, which is a prerequisite for remodeling and plasticity of individual synapses within a dendritic tree. In the fly visual system it was shown that synaptogenesis correlates with the appearance of mutual invaginations in photoreceptor terminals within a short time window (Rybak and Meinertzhagen, 1997). Using fluorescent markers for pre- and postsynaptic partners in a genetically controlled system (Chen et al., 2014), in combination with the visualization of spinules, correlated cellular activity of spinules and synaptic turnover could be demonstrated. In a longer perspective, using transgenic flies to block spinule fragmentation after synaptic activity, complemented by behavioral assays, will help us understand whether the trans-synaptic exchange of material through this novel mechanism has consequences for learning and memory.

## Author Contributions

RC, LG and JR conceived and designed the study and the outline for this perspective. Experiments and analyses were planned by JR and LG and performed by LG. LG, JR and RC interpreted and evaluated the data. Figures of this article were prepared by LG. LG, RC, JR and BSH wrote and discussed the manuscript. All authors critically revised the article.

## Conflict of Interest Statement

The authors declare that the research was conducted in the absence of any commercial or financial relationships that could be construed as a potential conflict of interest.

## Footnotes

^1^https://imagej.net/TrakEM2
