# Supplementary material for: Synaptic Spinules in the Olfactory Circuit of Drosophila melanogaster
Source: Front Cell Neurosci. 2018 Mar 27;12:86. doi: 10.3389/fncel.2018.00086 (PMC5880883; doi:10.3389/fncel.2018.00086)
Supplement: Supplementary file 1 [file Data_Sheet_1.docx]

**MATERIAL AND METHODS**

Flies

Flies of genotype *Orco-GAL4-UAS-GCaMP6.0* from the Bloomington *Drosophila* stock center (<https://bdsc.indiana.edu)> were reared on standard *Drosophila* food at 25°C and 70% humidity on a 12 hour/12 hour day/night cycle. Only females, 7-days old, were studied.

Transmission electron microscopy

Five flies were anesthetized, decapitated and dipped in 0.05% Triton X-100 in 0.1M Sørensen’s phosphate buffer, pH 7.3 and transferred to a droplet of freshly prepared ice-cooled fixative (2.5% glutaraldehyde and 2.0% paraformaldehyde in 0.1M Sørensen’s phosphate buffer, pH 7.3). The proboscis was cut off and the back of the head opened to allow quick fixation (after (Rybak et al., 2016)). Brains were dissected out and fixed for 2 hours. Samples were rinsed in ice-cooled Sørensen’s phosphate buffer. A region of interest (ROI), the DA2 glomerulus, inside the antennal lobe (AL) was marked with near-infrared laser branding according to (Bishop et al., 2011). Brains were then washed with 0.1M cacodylate buffer, pH 7.3 and post-fixed in 1% osmium tetroxide and 1% potassium ferrocyanide in cacodylate buffer for 2 hours. After rinsing with cacodylate buffer the brains were dehydrated with a graded acetone series, including an additional *en bloc* staining step in-between (brains were incubated in 1% uranyl acetate in 50% acetone for 30 minutes in the dark). The dehydrated brains were infiltrated with resin glycid ether 100 (Serva), embedded in pure resin and left in an oven at 60°C to polymerize during 48 h. After localization of the ROI with the help of semithin sections, serial ultrathin 50 nm sections were cut with a diamond knife (Ultra 45°, Diatome) on a Reichert Ultracut S (Leica) ultramicrotome, collected on single slot grids (2 x 1 mm), and imaged with a JEM 1400 (Jeol) operated at 80 kV. Digital micrographs were obtained with a Gatan Orius SC 1000 CCD camera (Gatan) with the Gatan Microscopy Suite software.

Focused ion beam-scanning electron microscopy (FIB-SEM)

The brains of two flies were imaged with FIB-SEM using a FEI Helios NanoLab G3 UC from Thermo Fisher. Brains were fixed, contrasted and embedded in resin blocks as above. Before imaging, the surface of the trimmed block was coated with a conductive layer of carbon to prevent charging artifacts. The laser brands used to landmark the ROI were visible on the surface of the block. Before serial imaging the ROI surface was protected via a local electron beam and an subsequent ion beam deposition of platinum using the gas injection system in order to remove the surrounding material and in order to reduce deposition. Serial images of the marked volume (the ROI) were generated by repeated cycles of milling and imaging, orthogonal to the block surface. The tissue was milled with a focused beam of gallium ions using FEI's Tomahawk ion column (accelerating voltage: 30 kV, beam current: 790 pA, milling steps: 20 nm). After each milling cycle the exposed surface, orthogonal to the block surface, was imaged with SEM detecting the back-scattered electrons with an in-column detector, the FEI’s Elstar electron column (3 kV accelerating voltage; 1.6 nA beam current; 10 µs dwell time). The Horizontal Field Width was 18 µm, and the resolution was 4.4 nm/pix in x and 4.4 nm/pix in y; (4096 x 3536). The milling/imaging cycles were controlled with the FEI Auto Slice and View operating 4.0 software (ThermoFisher).

Image alignment, 3D reconstruction and segmentation

FIB-SEM image stacks were aligned by maximizing the Pearson correlation coefficient of the central part of two consecutive images using template matching from the openCV library (<https://opencv.org>). Neuron reconstruction was done with TrakEM2, an ImageJ (Fiji) plugin (<https://imagej.net/TrakEM2>), performing a dense reconstruction according to (Rybak et al., 2016).

Different types of neurons were identified according to criteria (described more in detail in (Rybak et al., 2016;Tobin et al., 2017) based on morphology (branching pattern and diameter of single branches, among other criteria), total volume inside one glomerulus and ultrastructural details (as for example their synaptic inventory). These criteria allow a clear identification of uniglomerular projection neurons and olfactory receptor neurons whereas other cell types are more difficult to distinguish and are described here with the generic term “multigomerular neurons”. To visualize spinule shape, data were exported from the 3D viewer of ImageJ-Fiji either in .stl file format or as an Amira label field to Amira software for surface rendering using the Label Editor and SurfaceGen function of Amira (ThermoFisher). Digital data were then converted into a Skeleton graph by a simple threshold segmentation of the binary label file using the Autoskeleton function of Amira. Schematic drawings of spinules were done with the aid of Adobe Illustrator (Adobe). Single electron microscopy images were colorized with Adobe Photoshop (Adobe).

Quantification of spinules and Double Membrane Vesicles (DMVs)

Spinules and DMVs were quantified in randomly selected host cells of each of the three neuronal types mentioned above. Every neuronal protrusion penetrating another neuron was counted as a single spinule. Every cellular entrapment of similar shape and size but without connection to a spinule was counted as a DMV. Both counts reflect the total number spinules and of DMVs inside the reconstructed portion of one host cell. Data was shown as mean ± SEM. Statistical differences between groups were determined with one-way ANOVA test and the Tukey *post hoc* test using Prism 7 software (GraphPad).

Bishop, D., Nikic, I., Brinkoetter, M., Knecht, S., Potz, S., Kerschensteiner, M., and Misgeld, T. (2011). Near-infrared branding efficiently correlates light and electron microscopy. *Nat Methods* 8**,** 568-570.

Rybak, J., Talarico, G., Ruiz, S., Arnold, C., Cantera, R., and Hansson, B.S. (2016). Synaptic circuitry of identified neurons in the antennal lobe of *Drosophila melanogaster*. *J Comp Neurol* 524:1920-1956.

Tobin, W.F., Wilson, R.I., and Lee, W.-C.A. (2017). Wiring variations that enable and constrain neural computation in a sensory microcircuit. *Elife May 22;6 piie24838*
